# Effects of Noise Exposure on Systemic and Tissue-Level Markers of Glucose Homeostasis and Insulin Resistance in Male Mice

**DOI:** 10.1289/EHP162

**Published:** 2016-04-29

**Authors:** Lijie Liu, Fanfan Wang, Haiying Lu, Shuangfeng Cao, Ziwei Du, Yongfang Wang, Xian Feng, Ye Gao, Mingming Zha, Min Guo, Zilin Sun, Jian Wang

**Affiliations:** 1 Department of Physiology, Medical College, Southeast University, Nanjing, China; 2 Institute of Life Sciences, Southeast University, Nanjing, China; 3 Medical College, Southeast University, Nanjing, China; 4 Department of Endocrinology, Medical College, Affiliated ZhongDa Hospital of Southeast University, Nanjing, China; 5 School of Human Communication Disorders, Dalhousie University, Halifax, Nova Scotia, Canada

## Abstract

**Background:**

Epidemiological studies have indicated that noise exposure is associated with an increased risk of type 2 diabetes mellitus (T2DM). However, the nature of the connection between noise exposure and T2DM remains to be explored.

**Objectives:**

We explored whether and how noise exposure affects glucose homeostasis in mice as the initial step toward T2DM development.

**Methods:**

Male ICR mice were randomly assigned to one of four groups: the control group and three noise groups (N20D, N10D, and N1D), in which the animals were exposed to white noise at 95 decibel sound pressure level (dB SPL) for 4 hr per day for 20 successive days, 10 successive days, or 1 day, respectively. Glucose tolerance and insulin sensitivity were evaluated 1 day, 1 week, and 1 month after the final noise exposure (1DPN, 1WPN, and 1MPN). Standard immunoblots, immunohistochemical methods, and enzyme-linked immunosorbent assays (ELISA) were performed to assess insulin signaling in skeletal muscle, the morphology of β cells, and plasma corticosterone levels.

**Results:**

Noise exposure for 1 day caused transient glucose intolerance and insulin resistance, whereas noise exposure for 10 and 20 days had no effect on glucose tolerance but did cause prolonged insulin resistance and an increased insulin response to glucose challenge. Akt phosphorylation and GLUT4 translocation in response to exogenous insulin were decreased in the skeletal muscle of noise-exposed animals.

**Conclusions:**

Noise exposure at 95 dB SPL caused insulin resistance in male ICR mice, which was prolonged with longer noise exposure and was likely related to the observed blunted insulin signaling in skeletal muscle.

**Citation:**

Liu L, Wang F, Lu H, Cao S, Du Z, Wang Y, Feng X, Gao Y, Zha M, Guo M, Sun Z, Wang J. 2016. Effects of noise exposure on systemic and tissue-level markers of glucose homeostasis and insulin resistance in male mice. Environ Health Perspect 124:1390–1398; http://dx.doi.org/10.1289/EHP162

## Introduction

Noise, defined as “unwanted sound,” is one of the most widespread sources of environmental pollution. Beyond its effects on the auditory system, noise exposure has been associated with several adverse health outcomes, including hypertension, myocardial infarction, and impaired cognitive performance (Basner et al. 2014; Cheng et al. 2011; Cui et al. 2012; Fonseca et al. 2012). Thus, although the predominant concern with noise exposure is auditory damage, increasing attention has been paid to the nonauditory effects of noise.

Type 2 diabetes mellitus (T2DM) is a chronic, progressive disease characterized by relative insulin deficiency resulting from a combination of insulin resistance and decreased β-cell function. Although its exact etiology remains unclear, T2DM has been recognized as a quintessential multifactorial disease resulting from numerous interactions between genetic predisposition and environmental factors (Mensink 2005). The incidence of T2DM is increasing worldwide to such a dramatic extent that it has been called an “epidemic,” which, like many other global health crises, is largely attributable to unhealthy aspects in modern society, including environmental pollution.

Recently, exposure to residential traffic noise was found to be associated with an increased risk of T2DM in a Danish population-based cohort with > 57,000 participants (Sørensen et al. 2013), suggesting a possible effect of noise on the development of diabetes. Both observational and experimental studies in human and animal subjects have indicated that noise exposure (both acute and chronic) can act as a stressor to irritate the sympathetic nervous system and increase stress hormones (including catecholamines and glucocorticoids), which in turn may result in detrimental health outcomes (Basner et al. 2014; Kight and Swaddle 2011; Münzel et al. 2014; Pascuan et al. 2014). Furthermore, excess stress hormones such as corticosterone have been reported to be associated with the development of T2DM in human subjects and rodent models (Beaudry and Riddell 2012; Geer et al. 2014; Yuen et al. 2013; Zardooz et al. 2006). Thus, we hypothesize that noise exposure is a potential contributor to diabetes and that the stress response might serve as an underlying mechanism linking noise and T2DM. Considering the alarming increases in the prevalence of both T2DM (Zimmet et al. 2014) and noise pollution (Basner et al. 2014; Holzman 2014), it is important to explore the potential effects of noise exposure on the development of T2DM. In the present study, we evaluated the changes in mouse glucose homeostasis after broadband noise exposure at 95 dB SPL for 4 hr/day for 1, 10, or 20 consecutive days.

## Methods

### Animals

**Figure 1:**
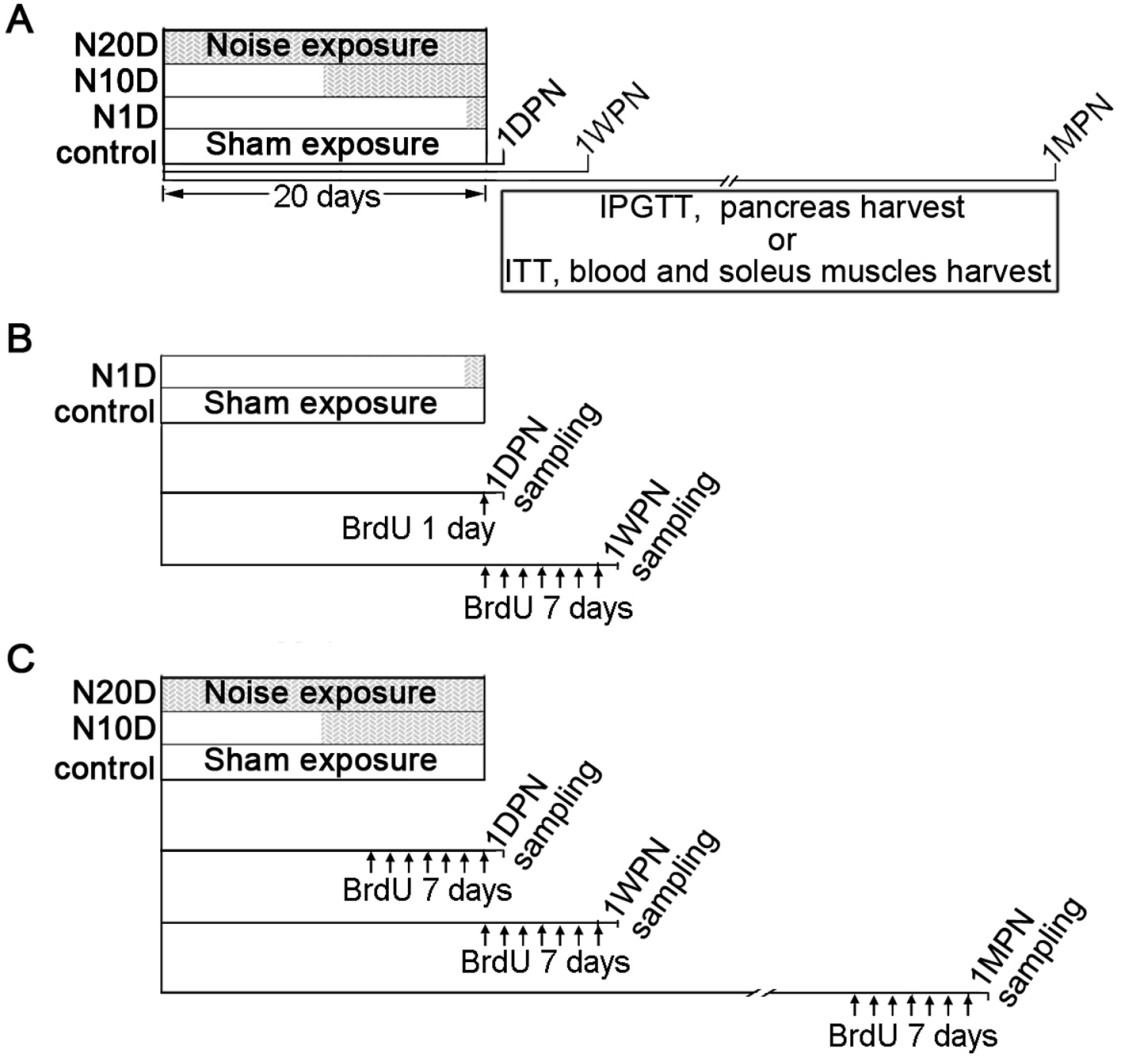
Experimental timeline. The animals were randomly assigned to four groups: the control group and three noise groups, in which the animals were subjected to 20 successive days, 10 successive days, or 1 day of noise exposure (N20D, N10D, and N1D, respectively) as indicated by the gray area within the 20-day period. Sham exposure, as indicated by the blank segments in the 20-day period, was performed for the animals in the control group for 20 days and for the animals in the N10D and N1D groups on the days when the noise exposure was not given. BrDU, bromodeoxyuridine; IPGTT, intraperitoneal glucose tolerance test; ITT, insulin tolerance test. (*A*) To study the effects of noise exposure on glucose metabolism, the animals in each group were further subdivided into three subgroups according to when the end-point evaluation was performed [1 day, 1 week, and 1 month after the final noise exposure (1DPN, 1WPN, and 1MPN, respectively)]. (*B*,*C*) To analyze β-cell proliferation, the mice were given 50 mg/kg BrdU intraperitoneally twice daily (every 12 hr, early in the morning and late in the evening) for 1 day [panel (*B*), only for the study of the N1D group at 1DPN] or for 7 successive days [panel (*C*)], and then the mice were sacrificed at the end time points.

Five-week-old wild-type ICR mice were obtained from the Qinglongshan Animal Center (Nanjing, China, SCXK(SU)2012-0008). To avoid uncertain sex-dependent differences, we included only male mice in the study. In total, 288 mice were subjects in this study (Table 1). All of the mice passed the Preyer reflex test (ear flick in response to a handclap) (Jero et al. 2001). The mice were housed in conventional cages with a 12-hr light/dark cycle (lights on at 0700 hours) and had free access to food (standard rodent chow diet, SHOOBREE; Xietong Organism, Jiangsu, China) and water. After a 1-week acclimation period, the animals were randomly assigned into one control group and three noise groups. The animals in the noise groups were exposed to a broadband noise at 95 decibel sound pressure level (dB SPL) for 4 hr per day between 0800 and 2000 hours for periods of 20 days (N20D group), 10 days (N10D group), or 1 day (N1D group) (Figure 1). The animals in each of the noise groups were further divided into three subgroups according to the end points when the assessments were performed: 1 day, 1 week, and 1 month after the final noise exposure (1DPN, 1WPN, and 1MPN, respectively). The mice in the control group were divided into the same subgroups according to the end time points and served as age-matched controls. The animals were treated humanely and with regard for alleviation of suffering. All of the animal procedures were approved by the University Committee for Laboratory Animals of Southeast University, China. Technicians involved in all tests were blinded to the exposure status.

**Table 1:** Numbers of animals used in each of the assessments.

Group	IPGTT, blood and pancreas harvest^*a*^	ITT, soleus muscle harvest^*b*^	Evaluation of β cell proliferation by BrdU injection for 1 day^*c*^	Evaluation of β cell proliferation by BrdU injection for 7 days^*d*^	Total
Control
1DPN	8	8	8	8	32
1WPN	8	8		8	24
1MPN	8	8		8	24
N1D
1DPN	8	8	8		24
1WPN	8	8		8	24
1MPN	8	8			16
N10D					
1DPN	8	8		8	24
1WPN	8	8		8	24
1MPN	8	8		8	24
N20D
1DPN	8	8		8	24
1WPN	8	8		8	24
1MPN	8	8		8	24
Total	96	96	16	80	288
Abbreviations: 1DPN, 1 day after termination of noise exposure; 1MPN, 1 month after termination of noise exposure; 1WPN, 1 week after termination of noise exposure; BrdU, bromodeoxyuridine; IPGTT, intraperitoneal glucose tolerance test; ITT, insulin tolerance test; N1D, mice exposed to noise for 1 day; N10D, mice exposed to noise for 10 days; N20D, mice exposed to noise for 20 days. Body weights of mice were measured 1 day before the experimental period and repeated at the end time points immediately before IPGTT or ITT. ^***a***^IPGTT on 16-hr fasted mice began at 0900 hours. After the test, the mice were given free access to food and water. Three to four hours later (i.e., 1400–1500 hours), blood (for corticosterone analysis) and pancreases were collected immediately after the mice were sacrificed by decapitation. ^***b***^ITT on 4-hr fasted mice began at 0900 hours. After the test, the mice were given free access to food and water. Three to four hours later (i.e., 1400–1500 hours), the mice were sacrificed by decapitation 20 min after insulin injection, and then soleus muscles were collected. ^***c***^The mice were given BrdU twice (0800 and 2000 hours) on the day of noise exposure. At 1DPN, the mice were sacrificed by decapitation, and the pancreases were collected. ^***d***^The mice were given BrdU twice (0800 and 2000 hours) daily for 7 successive days before the pancreases were harvested at 1DPN, 1WPN, and 1MPN.					

### Noise Exposure

Conditions of noise exposure at 95 dB SPL for 4 hr per day were chosen for this exploratory study of the potential role of noise on the development of diabetes because these conditions are comparable with the upper limit of the safety allowance presently recommended by the U.S. Occupational Safety and Health Administration (OSHA 1983). The animals were acclimatized to the setting of noise exposure for 30 min and were separated in metal net cages, awake, and unrestrained during noise exposure. Electrical Gaussian noise generated by a System III processor from Tucker-Davis Technologies (TDT, Alachua, FL, USA) was delivered to the speakers after power amplification. The acoustic spectrum of the sound was distributed primarily between 0.1 kHz and 20 kHz. The noise level was monitored using a 1/4-in. (6.35 mm) microphone linked to a sound level meter (Larson Davis 824; PCB Group, Inc., Depew, NY, USA). The conditions for sham exposure were identical except that the noise was not turned on.

### Auditory Brainstem Response (ABR)

Auditory function was assessed by measuring ABR thresholds at 1DPN. The animals were anesthetized with ketamine plus xylazine [40 mg/kg + 10 mg/kg, respectively, intraperitoneally (i.p.)], and their body temperatures were maintained at 38°C with a thermostatic heating pad. Three subdermal needle electrodes were used to record the ABR. Stimulus generation and response acquisition were performed using TDT hardware and software (BioSig).

### Intraperitoneal Glucose Tolerance Test

For an intraperitoneal glucose tolerance test (IPGTT), blood samples were taken from the tail veins of mice that had fasted for 16 hr before (0 min) and 5, 10, 30, 60, and 120 min after an i.p. injection of glucose (2 mg D-glucose/g body weight). The blood glucose level was measured using a portable glucose monitor (Bayer Contour; Bayer HealthCare LLC, Whippany, NJ), and the serum insulin level was measured with an insulin enzyme-linked immunosorbent assay (ELISA) kit (catalog number EZRMi-13k; Millipore, Billerica, MA, USA). The blood glucose and serum insulin levels recorded during the IPGTT were used to evaluate glucose tolerance and the insulin response to glucose challenge, respectively. The areas under the curves (AUCs) for blood glucose (AUC_IPGTT-glucose_) and serum insulin (AUC_IPGTT-insulin_) in response to glucose administration were calculated using SigmaPlot software (Systat Software, Inc., San Jose, CA). The blood glucose and serum insulin levels before glucose loading (0 min) were also used to represent the fasting blood glucose level (FBG) and fasting serum insulin level (FSI). A homeostasis model assessment of insulin resistance (HOMA-IR) was calculated as [FBG (in millimoles/liter) × FSI (in microunits/milliliter)]/22.5 (Andrikopoulos et al. 2008; Kiechl et al. 2013). Because there was no significant difference between the N1D group and the control in glucose tolerance and insulin response at 1WPN, the insulin response during the IPGTT of the N1D group at 1MPN was not studied.

### Insulin Tolerance Tests

Mice that had been fasted for 4 hr were injected i.p. with regular human insulin (Humulin, 0.75 U/kg body weight). The blood glucose concentrations were monitored before (0 min) and 15, 30, 45, 60, 90, and 120 min after insulin injection. The AUC for the blood glucose–time function (AUC_ITT-glucose_) was calculated using SigmaPlot software.

### Tissue and Blood Harvest

Trunk blood (for corticosterone analysis) was collected into dry tubes immediately after rapid decapitation. Following centrifugation at 4°C, the resulting serum was separated and stored at –80°C for later corticosterone level analysis. The pancreas was dissected, weighed, and fixed for immunohistochemistry by immersion in 4% (w/v) paraformaldehyde solution.

To collect the soleus muscle, mice were sacrificed by decapitation 20 min after i.p. injection of insulin (Humulin, 0.75 U/kg body weight). Then, the soleus muscles were dissected and immediately frozen in liquid nitrogen for immunoblotting analysis or were immersed in 4% (w/v) paraformaldehyde solution for immunohistochemistry.

To investigate the potential impact of noise on β-cell proliferation, additional mice were used in each group. Animals were given 50 mg/kg bromodeoxyuridine (BrdU) (catalog number B5002; Sigma-Aldrich, St. Louis, MO) i.p. twice daily (every 12 hr, early in the morning and late in the evening) for 7 successive days before the pancreases were harvested (Dor et al. 2004; Ellenbroek et al. 2013), except for the N1D group, to which BrdU was given only for 1 day before they were killed at 1DPN. We gave the 1-day BrdU injection to study the N1D group at 1DPN because the effects of noise on β-cell proliferation are likely to occur only during and/or shortly after noise exposure if they occur at all. Seven days of treatment with BrdU before harvesting the pancreases at 1DPN would label a large portion of β cells that had proliferated before the noise exposure and would therefore thin out the effects of the noise.

All of the sample collections were performed between 1400 and 1500 hours to avoid variations related to the circadian rhythm.

### Immunohistochemistry and Morphological Analysis

Cryosections (12 μm) of the pancreas and soleus muscles were cut and permeabilized with 0.01% TWEEN, blocked with 5% bovine serum albumin (BSA) for 1 hr at 37°C and then incubated overnight at 4°C with primary antibodies. Anti-GLUT4 (ab33780; Abcam, Cambridge, UK) was used for soleus muscle sections, and anti-insulin (SC-9168; Santa Cruz Biotechnology, Inc., Dallas, TX) was used for pancreas sections. After being washed with PBS, the sections were incubated with Alexa Fluor 488–conjugated chicken anti-rabbit antibodies (A-21441, Molecular Probes, Eugene, OR). The DNA fluorochrome 4´,6-diamidino-2-phenylindole (DAPI) was used to stain the nuclei. Images were acquired using an inverted fluorescence microscope (Olympus IX71, Tokyo, Japan) and were subjected to morphometric analysis using the ImageJ software package (Schneider et al. 2012). The processing and analysis were consistent between images.

For morphological examination of the pancreases, an average value of 8 to 10 sections, 200 μm apart from each other, was taken as a measure for the specimen. β-cell mass (milligrams per pancreas) was calculated by multiplying the relative insulin-positive area (the percentage of insulin-positive area over the total pancreas area) by the pancreas weight (Heit et al. 2006). The β-cell size was determined by dividing the total area of the β cells by the number of β cells (the number of DAPI-stained nuclei in insulin-positive cells). The relative insulin content in the β cells was calculated as the average intensity of insulin immunofluorescence per β cell after normalizing to the background. For β-cell proliferation assays, sections were double-stained for insulin and BrdU (anti-BrdU, catalog number ab6326; Abcam, Cambridge, UK). The ratio of insulin/BrdU double-positive cells to total insulin-positive cells in islets was calculated.

To determine the level and distribution of glucose transporter 4 (GLUT4) in the soleus muscle, six to eight consecutive sections (100 μm apart) from one specimen were stained for GLUT4. The relative GLUT4 content in the muscle cells was calculated using the average intensity of GLUT4 immunofluorescence per cell after normalizing to the background.

### Protein Preparation and Western Blot Analysis

Soleus muscle samples were homogenized in ice-cold radioimmunoprecipitation assay (RIPA) buffer (P0013C; Beyotime, Jiangsu, China) supplemented with complete protease inhibitor cocktail (Roche, Germany) and PhosSTOP (Roche, Germany). The protein concentrations in the supernatants obtained by centrifugation were measured usng a BCA protein assay kit (Pierce Biotechnology, Rockford, IL). The protein extracts (40 μg) for each preparation were separated by 10% sodium dodecyl sulfate–polyacrylamide gel electrophoresis (SDS-PAGE) and were then electrotransferred onto polyvinylidene fluoride (PVDF) (Millipore, Bedford, MA, USA). After blocking with 5% nonfat milk, the membranes were incubated with primary antibodies overnight at 4°C. The following antibodies were used: anti-AKT (catalog number 4685; Cell Signaling Technology, Beverly, MA, USA), anti-phospho-AKT Ser473 (catalog number 4058; Cell Signaling Technology), anti-GLUT4, and anti-GAPDH (catalog number AT0002; CMC-TAG, San Diego, CA, USA). The membranes were then washed and incubated with horseradish peroxidase–conjugated secondary antibodies. The protein bands were visualized using an ECL Kit (catalog number WBKLS0050; Millipore, Billerica, MA), and a densitometry analysis was performed using ImageJ. GAPDH was used as an internal control for quantitative analysis.

### Measurement of Corticosterone Levels

The serum corticosterone level was measured using a corticosterone ELISA kit (Nanjing Jiancheng Bioengineering Institute, Jiangsu, China) according to the manufacturer’s instructions. The results are expressed as nanograms/milliliter of serum.

### Statistics

The data were analyzed using SigmaPlot 12.5 (Systat Software, Inc., San Jose, CA, USA) and expressed as the mean ± standard error (SE). The normality of the raw data and residuals were tested with the Shapiro–Wilk test. Because of skewed distributions, the values of β-cell size, the relative insulin fluorescence intensity in β cells, and the percentages of proliferating β cells were logarithmically transformed (log_10_) for statistical analysis. Depending on the type of measurement, two-way or one-way analysis of variance (ANOVA) was performed with a focus on the effect of noise exposure (grouping). Differences among insulin or glucose concentration curves during the IPGTT or the ITT were evaluated using two-way repeated-measures ANOVAs against the factors of noise exposure (group) and time after the injection of glucose or insulin. Post-hoc pairwise comparisons between each noise group and the control group were performed (Tukey’s method) after a significant effect of noise exposure was obtained. Significance was assumed at *p* < 0.05.

## Results

### Effects of Noise Exposure on Hearing Threshold and Body Weight in Mice

The hearing threshold was estimated in ABR tests. A two-way ANOVA against noise and frequency revealed no significant threshold difference between the control and the noise-exposed animals even at 1DPN (see Figure S1), suggesting that the present noise exposures did not induce significant hearing loss. The mean body weight of every group increased over the entire duration of the study, but the growth patterns of the noise groups were different from those of the control group (see Figure S2). At 1DPN, the N10D and N20D groups had a significantly lower body weight than the control. However, no significant differences were observed between groups at 1WPN and 1MPN because of the increased growth rates in the noise groups after 1DPN.

### Effects of Noise Exposure on Glucose Tolerance and Insulin Response to the Glucose Challenge in Mice

**Figure 2:**
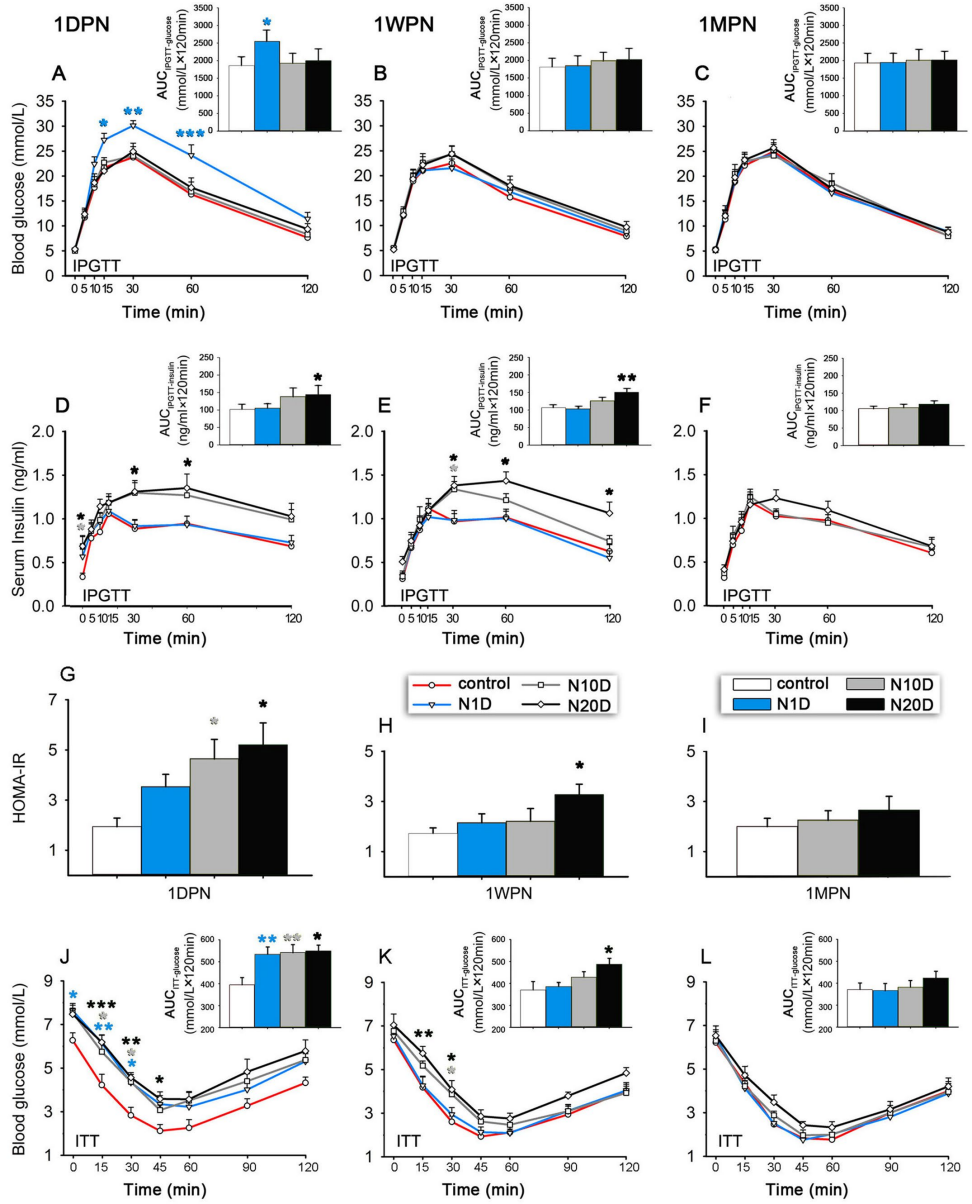
Effects of noise exposure on glucose homeostasis in mice. N1D, N10D, and N20D indicate mice that were exposed to 1, 10, and 20 days of noise, respectively. (*A*–*C*) Blood glucose levels recorded during the intraperitoneal glucose tolerance test (IPGTT) in 16 hr-fasted mice performed at 1 day, 1 week, and 1 month after termination of noise exposure (1DPN, 1WPN, and 1MPN, respectively). (*D*–*F*) The serum insulin levels recorded during the IPGTT in 16 hr-fasted mice were obtained at 1DPN, 1WPN, and 1MPN. (*G*–*I*) The homeostasis model assessment–insulin resistance (HOMA-IR) in 16 hr-fasted mice was estimated at 1DPN, 1WPN, and 1MPN. (*J*–*L*) The blood glucose levels during the insulin tolerance test (ITT) in 4 hr-fasted mice were assessed at 1DPN, 1WPN, and 1MPN. Insets are the corresponding results of the IPGTT and ITT analyzed by the area under the curve (i.e., AUC_IPGT-glucose_, AUC_IPGT-insulin_, or AUC_ITT-glucose_). Because the glucose tolerance and insulin response of the N1D group were the same as those of the controls at 1WPN, we did not measure the insulin response during the IPGTT of N1D at 1MPN. The values are presented as the means ± SEM of 8 mice per group. *p < 0.05, **p < 0.01 and ***p < 0.001 in post-hoc comparisons between each noise group and the controls after two-way repeated measures analysis of variance (ANOVA) or one-way ANOVA, showing a significant effect of noise.

Before glucose administration (0 min), all groups showed comparable FBG (Figure 2A–C). After glucose administration, blood glucose at 1DPN was significantly higher in the N1D group than in controls (Figure 2A), but there were no significant differences in any group at 1WPN or 1MPN (Figure 2B,C). Fasting blood insulin (0 min) was significantly higher in N10D and N20D mice at 1DPN than in controls (Figure 2D). The insulin response to glucose administration was significantly increased in N20D mice at 1DPN and in N10D and N20D at 1WPN, but it was not significantly different from controls in any group at 1MPN (Figure 2D–F). The insulin response during IPGTT was not tested at 1MPN in the N1D group because of the negative result at 1WPN.

### Effects of Noise Exposure on Insulin Sensitivity in Mice

At 1DPN, all noise groups exhibited decreased insulin sensitivity, indicated by increased HOMA-IR values (significant for N10D and N20D mice), significantly increased blood glucose levels during the insulin tolerance test, and larger AUC_ITT-glucose_ (Figure 2G,J) compared with controls. At 1WPN, the N1D group showed insulin sensitivity similar to that in the control group, whereas the N10D and N20D groups showed higher-than-control glucose levels at 15 and 30 min after insulin injection (Figure 2K). Although the N20D group exhibited a higher HOMA-IR value and a larger AUC_ITT-glucose_ at 1WPN (Figure 2H,K), no differences were observed between any of the groups at 1MPN (Figure 2I,L).

Multiple regression analysis showed that the factors of noise exposure duration and the interval between the end of exposure and assessment were significantly associated with the insulin sensitivity index (AUC_ITT-glucose_) (*r* = 0.516, *p* < 0.001). Pearson’s correlation analysis (see Figure S3) showed that AUC_ITT-glucose_ was positively correlated with noise exposure duration (*r* = 0.333, *p* < 0.001) but negatively correlated with the time between the end of exposure and outcome assessment (*r* = –0.395, *p* < 0.001), indicating that insulin resistance increased with the duration of noise exposure and then decreased over time after the noise exposure ended.

### Effects of Noise Exposure on Insulin Signaling in the Soleus Muscle

**Figure 3:**
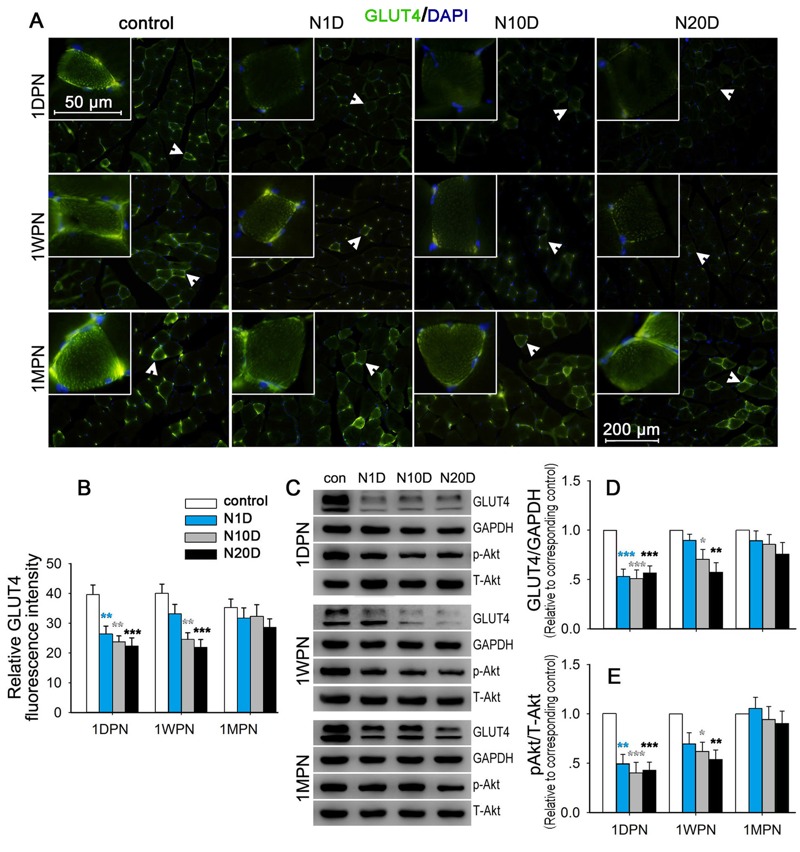
Effect of noise exposure on insulin signaling in the soleus muscle. N1D, N10D, and N20D indicate mice that were exposed to 1, 10, and 20 days of noise, respectively; 1DPN, 1WPN, and 1MPN refer to time intervals of 1 day, 1 week, and 1 month after termination of noise exposure, respectively. (*A*) Representative images of soleus sections subjected to GLUT4 (green) and 4′,6-diamidino-2-phenylindole (DAPI) (blue) immunofluorescence staining. The insets show higher magnifications of GLUT4-enriched cells signified by arrowheads. In the control mice, GLUT4 immunoreactivity was predominantly localized to the cell membrane and was concentrated in a granular pattern in the periphery of the cell. GLUT4 staining in the cell membrane and in the cytosolic fraction was notably weaker in the N10D and N20D mice at 1DPN and 1WPN than in the matched controls. All images were captured using a 20× objective. (*B*) The relative GLUT4 fluorescence intensity in the cells 20 min after exogenous insulin injection was normalized to the background and compared across groups. The results indicated a significant decrease in GLUT4 fluorescence intensity in all noise-exposed groups at 1DPN and in both the N10D and N20D groups at 1WPN. (*C*) The levels of GLUT4, GAPDH, phosphorylated Akt (p-Akt), and total Akt (T-Akt) were detected with immunoblotting, and representative Western blots are presented. (*D*,*E*) The levels of Akt phosphorylation and GLUT4 were quantified and normalized to age-matched controls. The average of each age-matched control group was set to 1. The values are presented as the means ± SEM of 8 mice per group. *p < 0.05, **p < 0.01 and ***p < 0.001 in post-hoc comparisons between each noise group and the control after a one-way analysis of variance (ANOVA), showing a significant effect of noise.

As illustrated in Figure 3A, the GLUT4 signal was predominantly localized near the nucleus and on the cell membrane in the control mice. The GLUT4 signal decreased in all three noise groups at 1DPN and in the N10D and N20D groups at 1WPN. Significant effects of noise were demonstrated at these two time points by the semi-quantitative analyses shown in Figure 3B.

The immunoblot results (Figure 3C–E) indicated that, compared with the control mice, GLUT4 levels and Akt phosphorylation were significantly reduced in all noise groups at 1DPN and in the N10D and N20D groups at 1WPN, whereas at 1MPN, there were no significant differences from controls for either outcome in any group.

### Effects of Noise Exposure on β-cell Morphology

**Figure 4:**
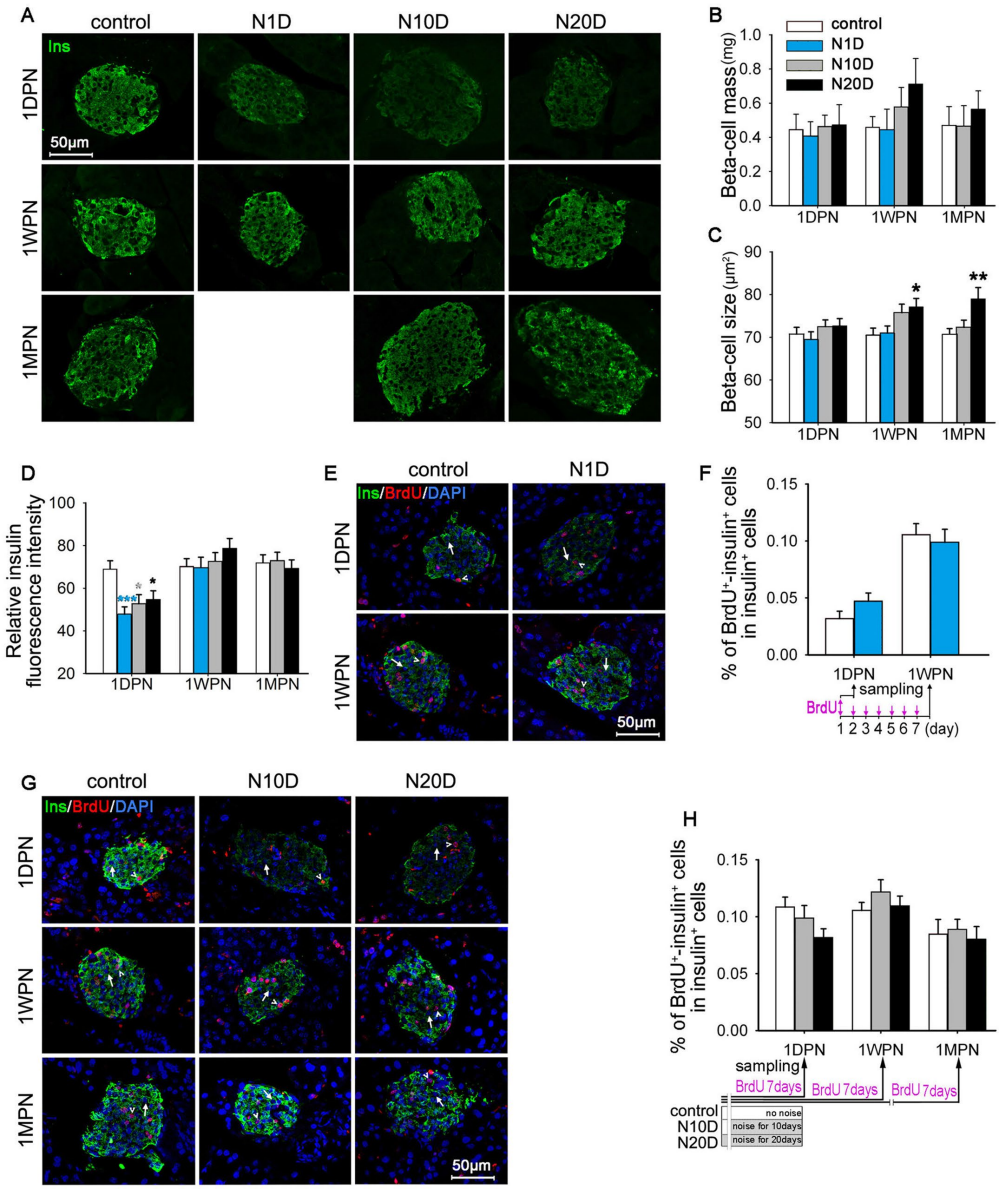
Effects of noise exposure on β cells in mice. N1D, N10D, and N20D indicate mice that were exposed to 1, 10, and 20 days of noise, respectively; 1DPN, 1WPN, and 1MPN refer to time intervals of 1 day, 1 week, and 1 month after termination of noise exposure, respectively. (*A*) Representative confocal images of pancreatic sections subjected to insulin (green) immunofluorescence staining. (*B–D*) Bar graph showing the β-cell mass (*B*), β-cell size (*C*), and relative insulin fluorescence intensity in β cells (*D*) in pancreatic sections. (*E*) Representative confocal images of pancreatic sections of N1D and age-matched control mice subjected to insulin (green), bromodeoxyuridine (BrdU) (red), and 4′,6-diamidino-2-phenylindole (DAPI) (blue) immuno­fluorescence staining. Examples of BrdU^–^-insulin^+^ cells are indicated by arrows, examples of BrdU^+^-insulin^+^ cells are indicated by arrowheads. (*F*) Bar graph showing the percentages of BrdU^+^-insulin^+^ cells among insulin^+^ cells in pancreatic sections from the N1D and age-matched control groups. (*G*) Representative confocal images of pancreatic sections from N10D, N20D, and age-matched control mice subjected to insulin (green), BrdU (red), and DAPI (blue) immunofluorescence staining. Examples of BrdU^–^-insulin^+^ cells are indicated by arrows, examples of BrdU^+^-insulin^+^ cells are indicated by arrowheads. (*H*) Bar graph showing the percentages of BrdU^+^-insulin^+^ cells among insulin^+^ cells in pancreatic sections from the N10D, N20D, and age-matched control groups. The timelines for BrdU below the *x*-axes of panels (*F*) and (*H*) showing the BrdU infusion protocols employed in corresponding studies. BrdU was administered twice daily for 7 successive days before the pancreases were harvested at the end time points (*F,H*), except for the N1D group, to which BrdU was given only for 1 day before they were killed at 1DPN (*F*). All images were captued using a 40× objective. The values are presented as the means ± SEM of 8 mice per group. *p < 0.05, **p < 0.01 and ***p < 0.001 in post-hoc comparisons between each noise group and the control group after one-way analysis of variance (ANOVA), showing a significant effect of noise. To correct for variance nonnormality, the data were log_10_ transformed for statistical analysis [panels (*C*,*D*,*F*,*H*)].

The pancreas weights of all animals tested at different time points were indistinguishable (data not shown). Although the mean β-cell mass of the N10D and N20D groups was higher than the mean in controls at 1WPN, there were no significant differences from controls for any exposure group at any time point (Figure 4B). β-cell size was significantly larger in the N20D mice than in controls at 1WPN and 1MPN (Figure 4C). A significantly decreased insulin signal intensity was observed in noise-exposed mice at 1DPN (Figure 4A,D).

To label the proliferating β cells, a subset of each group of mice was given BrdU i.p. for 7 successive days before the pancreases were harvested, except for the N1D group, to which BrdU was given for only 1 day before euthanasia at 1DPN to avoid the effects (if they existed) of 1 day of noise exposure on BrdU incorporation to be diluted by an additional 6 days of BrdU labeling before noise exposure. As illustrated in Figure 4E–H, there were no significant effects of noise exposure on BrdU incorporation into β cells, indicating no significant difference in β-cell proliferation.

We did not observe the β-cell morphology in the N1D group at 1MPN because there was no significant change in β-cell morphology and insulin response during the IPGTT of the N1D group at 1WPN.

### Analysis of Serum Corticosterone

**Figure 5:**
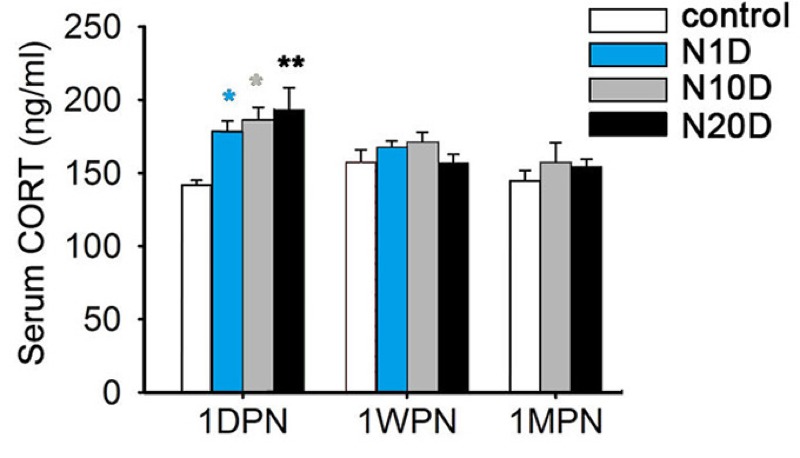
Effects of noise exposure on serum cortico­sterone (CORT) levels in mice. N1D, N10D, and N20D indicate mice that were exposed to 1, 10, and 20 days of noise, respectively; 1DPN, 1WPN, and 1MPN refer to time intervals of 1 day, 1 week, and 1 month after termination of noise exposure, respectively. The data are expressed as the means ± SEM for 8 mice in each group. *p < 0.05 and **p < 0.01 in post-hoc comparisons between each noise group and the control group after one-way analysis of variance (ANOVA), showing a significant effect of noise.

The serum corticosterone concentrations are shown in Figure 5; these results indicate significant effects of noise exposure only at 1DPN, which was when serum corticosterone was significantly elevated in every noise-exposed group compared with the control group.

## Discussion

Noise pollution is more severe and more widespread than ever before because of the rapid urbanization and industrialization of modern society (Basner et al. 2014; Holzman 2014). For our exploratory study of the potential effects and underlying mechanisms of noise exposure on glucose homeostasis, we exposed mice to 95-dB SPL noise for 4 hr per day, consistent with the regulatory limit for industrial noise exposure in the United States (OSHA 1983). However, noise exposure at or beyond this level may also occur in nonoccupational settings, such as at sporting events, loud concerts, and motorized sporting facilities, where peak sound levels of 110–117 A-weighted decibels (dBA) (equivalent to continuous exposure at 85–97 dBA) have been reported (Cranston et al. 2013; Ivory et al. 2014). The present data indicated that although noise exposure did not cause significant hearing loss (as indicated by comparable ABR thresholds in the control and noise-exposed groups), it did cause temporary insulin resistance that was prolonged with longer noise exposure and was consistent with the evidence of blunted insulin signaling in skeletal muscle. Our work suggests that noise exposure, particularly if prolonged, might be an environmental factor conferring individual vulnerability to T2DM.

T2DM is characterized by decreased insulin sensitivity in peripheral tissues and resulting perturbation of insulin secretion [American Diabetes Association (ADA) 2014]. The global epidemic of T2DM has been attributed to interactions between genetic susceptibility factors and increased exposure to environmental risk factors, although obesity is also likely to be an important contributor through its effects on insulin resistance (Kahn et al. 2006). In the present study, noise-exposed animals did not show a higher body weight than controls at 1DPN, 1WPN, or 1MPN, minimizing the possibility that body weight mediated the effects of noise on glucose metabolism. Conversely, body weights of the noise-exposed animals were lower than that of the control at 1DPN, although there were no differences in initial body weight among the groups. This result is in accord with those of previous reports stating that chronic noise exposure decreased body weight in rats (Alario et al. 1987) as a result of the increased energy expenditure caused by the noise-induced stress response (Alario et al. 1987; Kight and Swaddle 2011). After the cessation of noise exposure, the N10D and N20D groups exhibited accelerated weight gain, which resulted in the equal body weights of the noise groups at 1WPN and 1MPN. We did not collect information on food consumption and metabolism during the experiment; consequently, underlying mechanisms that might explain the weight changes observed in the present study remain elusive.

Skeletal muscle is the primary site of insulin-dependent glucose disposal (Nandi et al. 2004). In skeletal muscle, by binding and activating the insulin receptor, insulin phosphorylates and activates Akt, which in turn leads to the translocation of GLUT4 from intracellular locations to the plasma membrane, where it facilitates the transport of glucose into the cell (Brewer et al. 2014). Our finding of blunted Akt phosphorylation and decreased cell-surface GLUT4 in the skeletal muscle of both the N10D and N20D animals at 1DPN and 1WPN provides further evidence of insulin resistance in the noise-exposed animals.

Insulin resistance is the key pathological feature of T2DM, but insulin resistance by itself does not necessarily lead to diabetes. The optimal control of glucose metabolism also depends on the capacity for insulin synthesis and secretion by β cells (Cerf 2013; Tarabra et al. 2012). If the pancreatic β cells can sufficiently increase insulin release to overcome the reduced efficiency of insulin activity, normal glucose tolerance will be maintained (Kahn et al. 2006). In contrast, if insulin resistance culminates in the failure of islet β-cell compensation, overt diabetes will ultimately develop (Tiganis 2011). In the present study, noise-exposed animals had normal fasting blood glucose accompanied by higher fasting serum insulin and an elevated insulin response to glucose injection, consistent with a compensatory increase in pancreatic β-cell insulin secretion.

The process of β-cell compensation is a combination of an increase in glucose-stimulated insulin secretion and β-cell mass expansion (Rabhi et al. 2014). We compared the islet morphology of noise-exposed and control animals. No significant differences were observed in the β-cell mass or β-cell proliferation among the groups. The insulin fluorescence intensity assay revealed that along with the elevated fasting serum insulin level at 1DPN, the insulin-positive signal in β cells from the noise-exposed mice was significantly lower than that of the control, implying a functional up-regulation of insulin secretion at that time point. With time, the insulin signal in β cells recovered, but the β-cell size increased, particularly in the N20D mice at 1WPN and 1MPN, implicating β-cell compensation through hypertrophy, which may have contributed to the normal glucose tolerance exhibited in these animals.

Noise can induce a complex stress response involving the hypothalamo–pituitary–adrenal (HPA) axis (Samson et al. 2007). Previous studies have reported significant increases in plasma corticosterone levels during and after various noise exposures (Gannouni et al. 2013; Gonzalez-Perez et al. 2011; Manikandan et al. 2006). One day after noise exposure ended, plasma corticosterone levels were significantly higher in all noise groups than in controls regardless of the duration of noise exposure. This finding suggests that the noise induced stress reactions and that the animals did not adapt to the noise even after prolonged exposure (Samson et al. 2007).

Persistently elevated cortisol levels have been proposed to result in insulin resistance and are associated with the development of T2DM (Sjöstrand and Eriksson 2009). For example, subjects with a syndrome of cortisol excess (Cushing syndrome) showed increased insulin resistance (Nosadini et al. 1983). Insulin resistance has been induced by cortisol administration in man (Rizza et al. 1982) and in mice (van Donkelaar et al. 2014). Sleep deprivation is a neurobiologic and physiologic stressor that has been shown to increase cortisol concentrations and to decrease insulin sensitivity in humans (Donga et al. 2010; McEwen 2006; Spiegel et al. 1999). Furthermore, increased corticosterone was considered to be a contributor to increased insulin resistance induced by repeated restraint stress in rats (Zardooz et al. 2006). Therefore, a disturbance in a stress hormone such as cortisol caused by noise exposure might be a potential contributor to the development of insulin resistance observed in the present study.

However, even after noise exposure was terminated and serum corticosterone levels had reached control levels, insulin resistance continued for ≥ 1 month. This persistent decrease in insulin sensitivity might not be attributable merely to changes in blood glucocorticoid levels. Thus, our study strongly suggests that a second, somewhat delayed mechanism is involved in noise-induced insulin resistance.

## Conclusion

We found that noise exposure at levels consistent with those in industrial settings was associated with insulin resistance in male ICR mice. To our knowledge, this is the first study to identify this relationship in a laboratory setting. To further confirm the contribution of environmental noise pollution to diabetes risk, additional laboratory studies should be performed using lower noise levels that are consistent with those in urban environments. These findings should also be confirmed in female mice. Factors that may contribute to insulin resistance at the tissue level (e.g., inflammatory mediators, such as cytokines and chemokines, and reactive oxygen species, such as superoxide or hydroxyl radicals) (Sjöstrand and Eriksson 2009; Tiganis 2011), should also be investigated (through immunoblots, immunohistochemical methods, and quantitative polymerase chain reaction) to pinpoint the exact mechanisms of the diabetogenic effects of noise exposure.

## Supplemental Material

(485 KB) PDF

## References

[r1] ADA (American Diabetes Association) (2014). Diagnosis and classification of diabetes mellitus.. Diabetes Care.

[r2] Alario P, Gamallo A, Beato MJ, Trancho G (1987). Body weight gain, food intake and adrenal development in chronic noise stressed rats.. Physiol Behav.

[r3] Andrikopoulos S, Blair AR, Deluca N, Fam BC, Proietto J (2008). Evaluating the glucose tolerance test in mice.. Am J Physiol Endocrinol Metab.

[r4] Basner M, Babisch W, Davis A, Brink M, Clark C, Janssen S (2014). Auditory and non-auditory effects of noise on health.. Lancet.

[r5] Beaudry JL, Riddell MC (2012). Effects of glucocorticoids and exercise on pancreatic β-cell function and diabetes development.. Diabetes Metab Res Rev.

[r6] Brewer PD, Habtemichael EN, Romenskaia I, Mastick CC, Coster AC (2014). Insulin-regulated Glut4 translocation: membrane protein trafficking with six distinctive steps.. J Biol Chem.

[r7] Cerf ME 2013 Beta cell dysfunction and insulin resistance. Front Endocrinol (Lausanne) 4 37, doi:10.3389/fendo.2013.00037 23542897 PMC3608918

[r8] Cheng L, Wang SH, Chen QC, Liao XM (2011). Moderate noise induced cognition impairment of mice and its underlying mechanisms.. Physiol Behav.

[r9] Cranston CJ, Brazile WJ, Sandfort DR, Gotshall RW (2013). Occupational and recreational noise exposure from indoor arena hockey games.. J Occup Environ Hyg.

[r10] Cui B, Wu M, She X, Liu H (2012). Impulse noise exposure in rats causes cognitive deficits and changes in hippocampal neurotransmitter signaling and tau phosphorylation.. Brain Res.

[r11] Donga E, van Dijk M, van Dijk JG, Biermasz NR, Lammers GJ, van Kralingen KW (2010). A single night of partial sleep deprivation induces insulin resistance in multiple metabolic pathways in healthy subjects.. J Clin Endocrinol Metab.

[r12] Dor Y, Brown J, Martinez OI, Melton DA (2004). Adult pancreatic β-cells are formed by self-duplication rather than stem-cell differentiation.. Nature.

[r13] Ellenbroek JH, Töns HA, Westerouen van Meeteren MJ, de Graaf N, Hanegraaf MA, Rabelink TJ (2013). Glucagon-like peptide-1 receptor agonist treatment reduces beta cell mass in normoglycaemic mice.. Diabetologia.

[r14] Fonseca J, Martins-dos-Santos J, Oliveira P, Laranjeira N, Aguas A, Castelo-Branco N (2012). Noise-induced gastric lesions: a light and electron microscopy study of the rat gastric wall exposed to low frequency noise.. Arq Gastroenterol.

[r15] Gannouni N, Mhamdi A, Tebourbi O, El May M, Sakly M, Rhouma KB (2013). Qualitative and quantitative assessment of noise at moderate intensities on extra-auditory system in adult rats.. Noise Health.

[r16] Geer EB, Islam J, Buettner C (2014). Mechanisms of glucocorticoid-induced insulin resistance: focus on adipose tissue function and lipid metabolism.. Endocrinol Metab Clin North Am.

[r17] Gonzalez-Perez O, Chavez-Casillas O, Jauregui-Huerta F, Lopez-Virgen V, Guzman-Muniz J, Moy-Lopez N (2011). Stress by noise produces differential effects on the proliferation rate of radial astrocytes and survival of neuroblasts in the adult subgranular zone.. Neurosci Res.

[r18] Heit JJ, Apelqvist AA, Gu X, Winslow MM, Neilson JR, Crabtree GR (2006). Calcineurin/NFAT signalling regulates pancreatic β-cell growth and function.. Nature.

[r19] Holzman DC 2014 Fighting noise pollution: a public health strategy. Environ Health Perspect 122 A58, doi:10.1289/ehp.122-A58 24486882 PMC3915252

[r20] Ivory R, Kane R, Diaz RC (2014). Noise-induced hearing loss: a recreational noise perspective.. Curr Opin Otolaryngol Head Neck Surg.

[r21] Jero J, Coling DE, Lalwani AK (2001). The use of Preyer’s reflex in evaluation of hearing in mice.. Acta Otolaryngol.

[r22] Kahn SE, Hull RL, Utzschneider KM (2006). Mechanisms linking obesity to insulin resistance and type 2 diabetes.. Nature.

[r23] Kiechl S, Wittmann J, Giaccari A, Knoflach M, Willeit P, Bozec A (2013). Blockade of receptor activator of nuclear factor-κB (RANKL) signaling improves hepatic insulin resistance and prevents development of diabetes mellitus.. Nat Med.

[r24] Kight CR, Swaddle JP (2011). How and why environmental noise impacts animals: an integrative, mechanistic review.. Ecol Lett.

[r25] Manikandan S, Padma MK, Srikumar R, Jeya Parthasarathy N, Muthuvel A, Sheela Devi R (2006). Effects of chronic noise stress on spatial memory of rats in relation to neuronal dendritic alteration and free radical-imbalance in hippocampus and medial prefrontal cortex.. Neurosci Lett.

[r26] McEwen BS (2006). Sleep deprivation as a neurobiologic and physiologic stressor: allostasis and allostatic load.. Metabolism.

[r27] Mensink M (2005). Lifestyle intervention, glucose tolerance, and risk of developing type 2 diabetes mellitus.. Metab Syndr Relat Disord.

[r28] Münzel T, Gori T, Babisch W, Basner M (2014). Cardiovascular effects of environmental noise exposure.. Eur Heart J.

[r29] Nandi A, Kitamura Y, Kahn CR, Accili D (2004). Mouse models of insulin resistance.. Physiol Rev.

[r30] Nosadini R, Del Prato S, Tiengo A, Valerio A, Muggeo M, Opocher G (1983). Insulin resistance in Cushing’s syndrome.. J Clin Endocrinol Metab.

[r31] OSHA (Occupational Safety and Health Administration) (1983). Part 1910: Occupational Safety and Health Standards, Subpart G: Occupational Health and Environmental Control, Standard Number 1910.95: Occupational Noise exposure.. https://www.osha.gov/pls/oshaweb/owadisp.show_document?p_table=standards&p_id=9735.

[r32] Pascuan CG, Uran SL, Gonzalez-Murano MR, Wald MR, Guelman LR, Genaro AM (2014). Immune alterations induced by chronic noise exposure: comparison with restraint stress in BALB/c and C57Bl/6 mice.. J Immunotoxicol.

[r33] Rabhi N Salas E Froguel P Annicotte JS 2014 Role of the unfolded protein response in β cell compensation and failure during diabetes. J Diabetes Res 2014 795171, doi:10.1155/2014/795171 24812634 PMC4000654

[r34] Rizza RA, Mandarino LJ, Gerich JE (1982). Cortisol-induced insulin resistance in man: impaired suppression of glucose production and stimulation of glucose utilization due to a postreceptor detect of insulin action.. J Clin Endocrinol Metab.

[r35] Samson J, Sheeladevi R, Ravindran R, Senthilvelan M (2007). Stress response in rat brain after different durations of noise exposure.. Neurosci Res.

[r36] Schneider CA, Rasband WS, Eliceiri KW (2012). NIH Image to ImageJ: 25 years of image analysis.. Nat Methods.

[r37] Sjöstrand M, Eriksson JW (2009). Neuroendocrine mechanisms in insulin resistance.. Mol Cell Endocrinol.

[r38] Sørensen M Andersen ZJ Nordsborg RB Becker T Tjønneland A Overvad K 2013 Long-term exposure to road traffic noise and incident diabetes: a cohort study. Environ Health Perspect 121 217 222, doi:10.1289/ehp.1205503 23229017 PMC3569689

[r39] Spiegel K, Leproult R, Van Cauter E (1999). Impact of sleep debt on metabolic and endocrine function.. Lancet.

[r40] Tarabra E Pelengaris S Khan M 2012 A simple matter of life and death—the trials of postnatal beta-cell mass regulation. Int J Endocrinol 2012 516718, doi:10.1155/2012/516718 22577380 PMC3346985

[r41] Tiganis T (2011). Reactive oxygen species and insulin resistance: the good, the bad and the ugly.. Trends Pharmacol Sci.

[r42] van Donkelaar EL Vaessen KR Pawluski JL Sierksma AS Blokland A Cañete R 2014 Long-term corticosterone exposure decreases insulin sensitivity and induces depressive-like behaviour in the C57BL/6NCrl mouse. PLoS One 9 e106960, doi:10.1371/journal.pone.0106960 25310187 PMC4195581

[r43] Yuen KC, Chong LE, Riddle MC (2013). Influence of glucocorticoids and growth hormone on insulin sensitivity in humans.. Diabet Med.

[r44] Zardooz H, Zahedi Asl S, Gharib Naseri MK, Hedayati M (2006). Effect of chronic restraint stress on carbohydrate metabolism in rat.. Physiol Behav.

[r45] Zimmet PZ, Magliano DJ, Herman WH, Shaw JE (2014). Diabetes: a 21st century challenge.. Lancet Diabetes Endocrinol.

