# Supplementary material for: Effects of Noise Exposure on Systemic and Tissue-Level Markers of Glucose Homeostasis and Insulin Resistance in Male Mice
Source: Environ Health Perspect. 2016 Apr 29;124(9):1390–8. doi: 10.1289/EHP162 (PMC5010391; doi:10.1289/EHP162)
Supplement: (485 KB) PDF [file EHP162.s001.acco.pdf]

Environ Health Perspect
DOI: 10.1289/EHP162

Note to readers with disabilities: *EHP* strives to ensure that all journal content is accessible to all readers. However, some figures and Supplemental Material published in *EHP* articles may not conform to 508 standards due to the complexity of the information being presented. If you need assistance accessing journal content, please contact ehp508@niehs.nih.gov. Our staff will work with you to assess and meet your accessibility needs within 3 working days.

Supplemental Material

Effects of Noise Exposure on Systemic and Tissue-Level Markers of Glucose Homeostasis and Insulin Resistance in Male Mice

Lijie Liu, Fanfan Wang, Haiying Lu, Shuangfeng Cao, Ziwei Du, Yongfang Wang, Xian Feng, Ye Gao, Mingming Zha, Min Guo, Zilin Sun, and Jian Wang

Table of Contents

Figure S1. ABR threshold comparison across the groups. The acoustic stimulus consisted of 10-ms tone bursts presented at a rate of 21.1/sec with frequencies of 2, 4, 8, 12, 16, 32, and 48 kHz. The stimuli were fed to a broadband speaker (MF1 from TDT) that was placed 10 cm away from the pinna of the animal. Three subdermal needle electrodes were used to record the ABR. At each frequency, the sound intensity was decreased in 5-dB steps from 90 dB SPL down to the threshold, which was defined as the lowest sound level at which a repeatable waveform of peak 3 or the 3–4 complex was visible. N=8 in each group.

DOI: 10.1289/EHP162

Figure S2. Effects of noise exposure on body weight (A) and body weight gain (fold change vs 1DPN) after the cessation of exposure (B). Body weights were measured using a digital top-loading balance (±0.01 g, BL-2200H, Shimadzu Corporation, Japan). The measurement was performed one day before the experimental period (served as the baseline) and repeated at the end time points immediately before IPGTT or ITT. To avoid food intake-induced variation, only data from 16-h fasted mice were used for statistic analysis on the effects of noise exposure on body weight and body weight gain. Post-hoc pairwise comparisons against age-matched controls performed after a two-way ANOVA (A) and one-way ANOVA at the two time points showed a significant effect of noise exposure (B). *p<0.05, **p<0.01 and ***p<0.001.

Figure S3 Relationships between insulin sensitivity and the duration of noise exposure (A) and the delay after noise exposure (B). Pearson's correlation analysis was used to assess the relationship between $AUC_{ITT\text{-}glucose}$ and the different variables. $AUC_{ITT\text{-}glucose}$ was positively correlated with the duration of noise exposure (r =0.333, P = 0.000922) but negatively correlated to the time between the end of exposure and outcome assessment (r = –0.395, P =0.0000685), suggesting a trend of an increase in insulin resistance with prolonged noise exposure and a recovery in insulin sensitivity with increasing delay after noise exposure.

DOI: 10.1289/EHP162

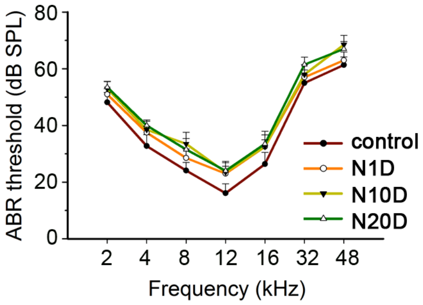

Figure S1. ABR threshold comparison across the groups. The acoustic stimulus consisted of 10-ms tone bursts presented at a rate of 21.1/sec with frequencies of 2, 4, 8, 12, 16, 32, and 48 kHz. The stimuli were fed to a broadband speaker (MF1 from TDT) that was placed 10 cm away from the pinna of the animal. Three subdermal needle electrodes were used to record the ABR. At each frequency, the sound intensity was decreased in 5-dB steps from 90 dB SPL down to the threshold, which was defined as the lowest sound level at which a repeatable waveform of peak 3 or the 3–4 complex was visible. N=8 in each group.

DOI: 10.1289/EHP162

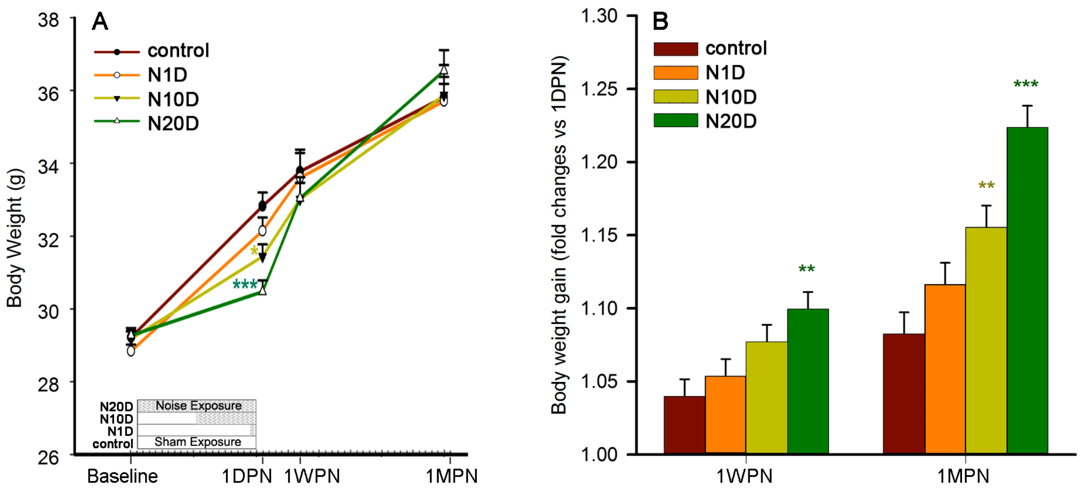

Figure S2. Effects of noise exposure on body weight (A) and body weight gain (fold change vs 1DPN) after the cessation of exposure (B). Body weights were measured using a digital top-loading balance (±0.01 g, BL-2200H, Shimadzu Corporation, Japan). The measurement was performed one day before the experimental period (served as the baseline) and repeated at the end time points immediately before IPGTT or ITT. To avoid food intake-induced variation, only data from 16-h fasted mice were used for statistic analysis on the effects of noise exposure on body weight and body weight gain. Post-hoc pairwise comparisons against age-matched controls performed after a two-way ANOVA (A) and one-way ANOVA at the two time points showed a significant effect of noise exposure (B). **p*<0.05, ***p*<0.01 and ****p*<0.001.

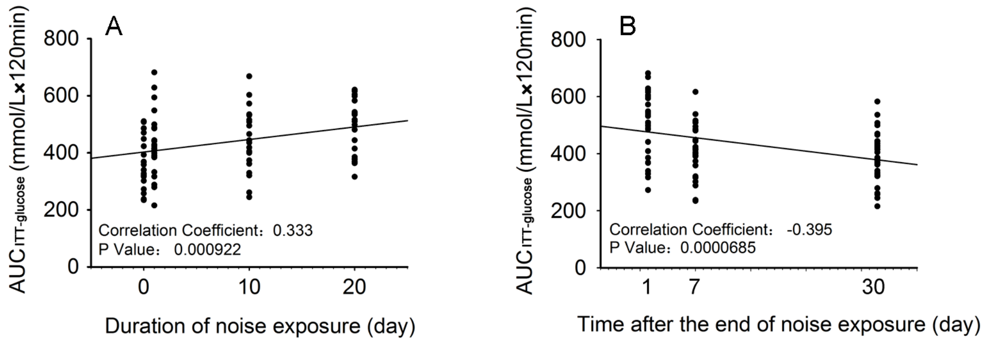

Figure S3 Relationships between insulin sensitivity and the duration of noise exposure (A) and the delay after noise exposure (B). Pearson's correlation analysis was used to assess the relationship between $AUC_{ITT\text{-}glucose}$ and the different variables. $AUC_{ITT\text{-}glucose}$ was positively correlated with the duration of noise exposure (r =0.333, P = 0.000922) but negatively correlated to the time between the end of exposure and outcome assessment (r = –0.395, P =0.0000685), suggesting a trend of an increase in insulin resistance with prolonged noise exposure and a recovery in insulin sensitivity with increasing delay after noise exposure.
